# Relevance of Hypersexual Disorder to Family Medicine and Primary Care as a Complex Multidimensional Chronic Disease Construct

**DOI:** 10.1155/2013/519265

**Published:** 2013-08-27

**Authors:** Liesbeth Borgermans, Bert Vrijhoef, Jan Vandevoorde, Jan De Maeseneer, Johan Vansintejan, Dirk Devroey

**Affiliations:** ^1^Department of Family Medicine, Vrije Universiteit Brussel (VUB), Laarbeeklaan 103, 1090 Brussels, Belgium; ^2^Scientific Center for Care and Welfare, Tilburg University, 5000 LE Tilburg, The Netherlands; ^3^Department of Family Medicine and Primary Care, University Ghent, 9000 Ghent, Belgium

## Abstract

Hypersexual disorder (HD) is not defined in a uniform way in the psychiatric literature. In the absence of solid evidence on prevalence, causes, empirically validated diagnostic criteria, instruments for diagnosis, consistent guidelines on treatment options, medical and psychosocial consequences, and type of caregivers that need to be involved, HD remains a controversial and relatively poorly understood chronic disease construct. The role of family medicine in the detection, treatment, and followup of HD is not well studied. The purpose of this paper is to describe the complexity of HD as a multidimensional chronic disease construct and its relevance to family medicine and primary care.

## 1. Introduction

Hypersexual disorder (HD) [1], also previously known as out-of-control sexual behavior, impulse control disorders [2], sexual addiction, sexual compulsivity, and sexual desire dysregulation [3], is not defined in a uniform way in the psychiatric literature. Although the clinical presentation of the disorder is varied, HD is characterized by an increased frequency and intensity of sexually motivated fantasies, arousal, urges, and enacted behavior in association with an impulsivity component over a period of ≥6 months [4]. It is estimated that 6% of the general population in the USA is afflicted [5], whereas to our knowledge no reliable estimates on the prevalence of HD in Europe are available.

The usefulness of the term HD depends upon the degree to which it can be defined, measured, and distinguished from other psychiatric disorders and nonpathological sexual behaviour [6]. Diagnostic and Statistical Manual of Mental Disorders, Fifth Edition (DSM-V), criteria for HD have been proposed by the Work Group on Sexual and Gender Identity Disorders to capture symptoms reported by patients seeking help for out-of-control sexual behavior [7, 8]. HD is nowadays not included in DSM-V as a distinct disorder as it requires more research and evidence to illuminate the cause, diagnosis, and treatment. In the absence of solid evidence on prevalence, causes, empirically validated diagnostic criteria, instruments for diagnosis, consistent guidelines on treatment options, medical and psychosocial consequences, and type of caregivers that need to be involved, HD remains a controversial and relatively poorly understood chronic disease construct. Whether or not HD will be considered as a distinct disorder in the next Diagnostic and Statistical Manual of Mental Disorders, we think it is important to outline its inherent complexity. In the light of the growing number of chronic neuropsychiatric disorders, including HD [9], an increased understanding is needed on complexity in HD and the role of family physicians and primary care in this disorder. This work further builds on research we conducted on complexity in chronic care delivery [10–14] and integrative family medicine [15, 16]. This paper is the first in a series of articles that will cover complexity in chronic care using different chronic diseases as an example.

## 2. Methods

A narrative review method was used to document the complexity and multidimensionality of HD, excluding HD in Parkinson's and Alzheimer's diseases, rare neurological/sleep related disorders (e.g., Kleine-Levin syndrome), brain injury (e.g., stroke), schizophrenia, and HD as a result of chirurgical interventions, since these afflictions are recognized as being mostly treated by medical specialists. 

## 3. Results

### 3.1. HD as a Multidimensional Chronic Disease Construct

Chronic diseases have been defined by the WHO as requiring “ongoing management over a period of years or decades” [17]. Chronic illness is usually characterized by complex causality, multiple risk factors, a prolonged course of illness, functional impairment or disability, and, sometimes, the unlikelihood of cure [18]. To document the complexity and multidimensionality of HD as a chronic disease construct, we further build on four major and interrelated components of complexity in chronic care that have been described by Borgermans et al. [10]. These components are (1) case (patient) complexity; (2) complexity of the care processes provided; (3) the complexity of quality assessment; and (4) complexity at the health system level. Each of these components represents a range of elements that contribute to the picture of complexity in chronic care. 

### 3.2. Case Complexity

The Vector Model of Complexity (VMC) [19] is a useful model to describe case complexity in patients with HD. The vector model proposes that the complexity of an individual patient arises out of interactions between different domains: biology, genetics, socioeconomics, environment, culture, behavior, and the health system. In a chronic condition such as HD, these “forces” are not easily discerned. 

#### 3.2.1. Biological Axis

Cooccurring psychiatric disorders in patients with HD contribute to the overall complexity along the biological vector of the VMC. Examples are bipolar disorder, depression, anxiety, attention deficit hyperactivity disorders, personality disorders (e.g., narcissistic personality disorder or borderline personality disorder), and paraphilias (exhibitionism, voyeurism, and masochism/sadism) [20, 21]. There is also a high comorbidity between HD and other addictive behaviors [22]. The latter can be explained since addictions are mediated by complex neural mechanisms that involve multiple brain circuits and neuroadaptive changes in a variety of neurotransmitter and neuropeptide systems [23]. The implication of the dopamine (DA) in reward mechanisms has been described by Blum and colleagues [24], amongst others. DA has become to be known as the “pleasure hormone” and/or the “antistress hormone.” A consensus of the literature suggests that when there is a dysfunction in the brain reward cascade, the brain of that person requires a DA fix to feel good. This trait leads to multiple drug-seeking behavior. Therefore lack of specific DA receptors causes individuals to have a high risk for multiple addictive, impulsive, and compulsive behavioral propensities, such as sex addiction, severe alcoholism, cocaine, heroin, marijuana, and nicotine use, glucose bingeing, pathological gambling, and chronic violence, amongst others [24]. Some authors consider HD as a clinical subset of the Reward Deficiency Syndrome (RDS) since it has similar neurogenetic polymorphisms to other addictions [25]. 

#### 3.2.2. Genetic Axis

Complexity in HD is introduced along the genetic axis as genetic predisposition towards HD is assumed [26, 27]. Social scientists now reflect on the human genome to explain human condition including sexuality and pathology [28]. However, the genetic predictors available are few in number and account for only a small portion of the genetic variance in liability and have not been integrated into clinical nosology or care [29].

#### 3.2.3. Environment/Socioeconomic Axis

Complexity in HD is introduced along the environment/socioeconomic axis with a growing number of studies to document the important relationship between parenting behavior and psychopathology [30, 31]. Previous studies consistently identified HD to be a dysfunctional child, teenager, or adult response to early attachment disorders, abuse, and trauma [32–35]. Dysfunctional family dynamics (substance abuse, addiction, parents who were emotionally unavailable, uncaring, or rigid in their parenting style) are associated with HD [36]. Other environmental and childhood determinants to the development of HD are socioeconomic position and living in a major city area, amongst others [20, 34].

#### 3.2.4. Behavioral Axis

Complexity in HD is also introduced along the behavioral axis as HD has considerable implications on daily life. Sexual behaviors in patients suffering from HD are intended to reduce anxiety and other dysphoric affects (e.g., shame and depression). In this sense HD is considered an escape from painful or unpleasant emotions and a reaction to stress [4]. A recent and large scale study showed that functional impairment in at least one life area was specified by the majority of the respondents including interference with social or occupational functioning (e.g., sexual harassment, surfing the Internet for porn rather than working), and most participants reported impairment regarding partner relationships [37]. 

### 3.3. Care Complexity

The complexity of clinical care for patients with HD is based on the number and the types of interventions that are required as well as the number of disciplines that is required to make major interventions. It is not well documented which type of professional caregivers should ideally be involved in the detection, treatment, and followup of patients with HD. Management of HD as a chronic medical condition is a complex process and requires coordinated action between healthcare providers of different kinds and patients. Unlike the psychiatric professional who sees patients, referred or not, who accept the diagnosis and the need for treatment, the family physician has to identify HD that is frequently obscured by patient reluctance to acknowledge the problem or by physical symptoms that mask the underlying problem [38]. As a consequence, the burden of HD is likely to be underestimated as a consequence of the inadequate recognition of the connection between mental and physical health [39]. Patient-based symptom and diagnosis severity measures that are available in the DSM-V for other mental disorders would allow for more individualized diagnosis than was hitherto possible.

Care complexity in HD is even more important since there is limited evidence for the treatment of hypersexual disorder [40, 41]. Moreover, treatment options are complicated since the population of individuals reporting hypersexual behavior is heterogeneous [37]. Treatment may include a combination of psychotherapy (including group and family therapy), medication, and support groups. The use of peer specialists (PSs)—individuals with serious mental illness who use their experiences to help others with serious mental illness—is increasing in primary Axis 1 psychiatric disorders [42]. In contrast to the support for many other psychiatric disorders, sexual disorders are not as well supported; for example, there is a lack of self-help materials, systematic provision of information, and support groups for patients, which may be related to a lower empowerment of this patient population.

Overall, controlled studies are warranted in order to establish clear guidelines for treatment of HD [22].

### 3.4. Quality Assessment Complexity

The complexity of quality assessment is reflected by the lack of tools at the present time that can assess the quality of care delivered to patients with HD. To our knowledge no specific quality indicators at the structure, process, or outcome level of care for patients with HD exist, which is in part a consequence of the lack of research on (the construct of) HD. In addition, publications that describe approaches to patient involvement in quality indicator development are scarce [43] and to our knowledge nonexisting for patients with sexual disorders [44].

### 3.5. (Health) System Complexity

Health system complexity is of relevance to health seeking behaviour of patients with HD. There is an abundance of studies on health seeking behaviour highlighting the importance of health system characteristics and their influence on an individual's behaviour at a given time and place [45, 46]. These influences include the financing of care, access to care, coordination mechanisms, existing stigma on mental health, and values and norms, amongst others. There is a tendency in the development of quality improvement programmes for chronic conditions to incorporate knowledge about health seeking behaviour into health service delivery strategies in a way which is sensitive to the local dynamics of the community [47]. This phenomenon of context dependence has led to calls for tailoring interventions to the cultural background of patients or the adaptation of practice guidelines for healthcare professionals. 

### 3.6. Relevance of HD as a Multidimensional Chronic Disease Construct to Family Medicine and Primary Care

We have outlined the importance of case, care, quality assessment, and health system complexity in patients with HD. This analysis is meant to contribute to the debate on the complexity of HD as a multidimensional chronic disease construct and the role of family medicine and primary care in the detection, counselling, and treatment of patients with HD. 

While psychiatric professionals are an essential element of the total health care continuum, the majority of patients with HD, as with other mental health issues, will continue to access the health care system through family physicians [48]. In many respects family medicine represents the unification of the psychiatric and physical models of illness [38]. The need over the principal care responsibility of family physicians for patients with HD is supported by concerns that the predominance of specialty physicians reduces access for vulnerable populations and increases the total cost of medical care. Other and equally important reasons in favor of the principal care responsibility of family physicians are the provision of accessible, continuous, coordinated, integrative, and comprehensive care. These are core attributes of primary care as defined by the Institute of Medicine [49]. 

A key barrier to seeking help in patients with HD is the stigma attached to mental health issues and sexual disorders in particular [50, 51]. Building on trust, family physicians can help their patients to express concerns related to HD, and in doing so fighting the stigma that rests upon sexual disorders. In this sense the clinician's ability to develop and utilize interactional relationships and resources needed to recognize and treat a person with HD is key to HD care in primary care settings. In this context it is important to recognize that family practice is a three-dimensional specialty incorporating the dimensions of knowledge, skill, and process [52]. While knowledge and skill should be shared with other specialties, by means of integrated networks [53], the family practice process is unique. At the center of this process is the patient-physician relationship with the patient viewed in the context of the family, which is essential in patients with HD. Our analysis on complexity in HD highlights the importance of comprehensiveness in this disorder. Comprehensiveness is the ability of the family physician to address a broad range of patient problems, whether or not the conditions are within the traditional domain of the specialty in which the physician is trained. In this sense, the scope of family medicine is not defined by diagnoses or procedures but by human needs. The recognition, integration, and prioritization of multiple concerns and the synthesis of solutions are critical clinical competencies that are essential to patients with HD. Referral to psychiatrists, psychiatric nurses, counselors, or psychologists, either attached to the practice or in other organizations, is essential both to meet the patient's needs and to establish an integrated care network where responsibilities and tasks are shared. An effective response to the health needs of those with HD will especially require family physicians and psychiatrists to expand their collaborative efforts and knowledge of each other's practices and treatments. 

Another component of the principal care responsibility is ensuring that persons vulnerable to developing HD receive preventive interventions. The continuity of care inherent in family medicine makes early recognition of possible problems [38]. Because family physicians treat the whole family, they are often better able to recognize problems and provide interventions in the family system. Since there is important evidence that adverse life events during childhood including, for example, neglect, maltreatment, and sexual abuse, are associated with HD and other adverse medical, psychological, behavioural, and socioeconomic outcomes in adulthood [54–56], the disorder is of particular importance to family physicians. HD that was once thought to affect only adults is now also known to affect children and adolescents which makes the relevance of this disorder even more important to family medicine although diagnostic criteria often require adaptation in order to be developmentally appropriate. 

Our analysis on complexity in HD has shown that HD is also of specific relevance to family physicians because of the related adverse psychosocial and medical consequences, such as unplanned pregnancy, pair-bond dysfunction, marital separation, addictive behaviors, and HIV [22, 57].

## 4. Conclusion

Each of the aforementioned components (case, care, quality assessment, and health systems complexity) represents a range of elements that contribute to the picture of complexity in HD as a multidimensional disease construct. We emphasis the need to reflect upon the principal care responsibility of family physicians in the prevention, detection, and counselling of patients with HD supported by specialists and other (primary) health care providers by means of integrated care networks. Further research and debate are needed on both HD as a multidimensional chronic disease construct and its relevance to family medicine especially for what concerns the application of diagnostic criteria, treatment, and referral guidelines. Enhanced diagnostic accuracy and referral must be connected to structured programmes that provide effective treatment, patient empowerment, regular patient followup, monitoring of treatment adherence, and the use of mental health specialists for the more severely ill patients.

## References

[1] Kafka MP, Hennen J (2003). Hypersexual desire in males: are males with paraphilias different from males with paraphilia-related disorders?. *Sexual Abuse*.

[2] Bancroft J (2008). Sexual behavior that is “Out of Control”: a theoretical conceptual approach. *Psychiatric Clinics of North America*.

[3] Bancroft J (1999). Central inhibition of sexual response in the male: a theoretical perspective. *Neuroscience and Biobehavioral Reviews*.

[4] Echeburua E (2012). Does really sex addiction exist?. *Adicciones*.

[5] Black DW (2000). The epidemiology and phenomenology of compulsive sexual behavior. *CNS Spectrums*.

[6] Baumelou A, Marcelli F, Robin G, Rigot JM (2013). Normal sexuality and sexual disorders. *Revue du Praticien*.

[7] Kafka MP (2010). Hypersexual disorder: a proposed diagnosis for DSM-V. *Archives of Sexual Behavior*.

[8] Reid RC, Carpenter BN, Hook JN (2012). Report of findings in a DSM-5 field trial for hypersexual disorder. *Journal of Sexual Medicine*.

[9] World Health Organisation (2008). *The Global Burden of Disease: 2004 Update*.

[10] Borgermans L, de Maeseneer J, Wollersheim H, Vrijhoef B, Devroey DA theoretical lens for revealing the complexity of chronic care.

[11] de Maeseneer J, Roberts RG, Demarzo M (2011). Tackling NCDs: a different approach is needed. *The Lancet*.

[12] de Maeseneer J, Boeckxstaens P (2012). James Mackenzie Lecture 2011: multimorbidity, goal-oriented care, and equity. *British Journal of General Practice*.

[13] de Maeseneer J, Boeckxstaens P (2011). Care for noncommunicable diseases (NCDs): time for a paradigm-shift. *World Hospitals and Health Services*.

[14] Gress S, Baan CA, Clanan M (2009). Co-ordination and management of chronic conditions in Europe: the role of primary care—position paper of the European forum for primary care. *Quality in Primary Care*.

[15] de Maeseneer JM, van Driel ML, Green LA, van Weel C (2003). The need for research in primary care. *The Lancet*.

[16] van Weel C, de Maeseneer J, Roberts R (2008). Integration of personal and community health care. *The Lancet*.

[17] World Health Organization (2013). Noncommunicable diseases country profiles 2011. *WHO Global Report*.

[18] Warsi A, Wang PS, LaValley MP, Avorn J, Solomon DH (2004). Self-management education programs in chronic disease: a systematic review and methodological critique of the literature. *Archives of Internal Medicine*.

[19] Safford MM, Allison JJ, Kiefe CI (2007). Patient complexity: more than comorbidity. The vector model of complexity. *Journal of General Internal Medicine*.

[20] Långström N, Hanson RK (2006). High rates of sexual behavior in the general population: correlates and predictors. *Archives of Sexual Behavior*.

[21] Scanavino MD, Ventuneac A, Abdo CH (2013). Compulsive sexual behavior and psychopathology among treatment-seeking men in Sao Paulo, Brazil. *Psychiatric Research*.

[22] Garcia FD, Thibaut F (2010). Sexual addictions. *American Journal of Drug and Alcohol Abuse*.

[23] Cui C, Noronha A, Morikawa H (2013). New insights on neurobiological mechanisms underlying alcohol addiction. *Neuropharmacology*.

[24] Blum K, Braverman ER, Holder JM (2000). Reward deficiency syndrome: a biogenetic model for the diagnosis and treatment of impulsive, addictive, and compulsive behaviors. *Journal of Psychoactive Drugs*.

[25] Blum K, Oscar-Berman M, Barh D, Giordano J, Gold M (2013). Dopamine genetics and function in food and substance abuse. *Journal of Genetic Syndromes & Gene Therapy*.

[26] Kendler KS (2013). What psychiatric genetics has taught us about the nature of psychiatric illness and what is left to learn. *Molecular Psychiatry*.

[27] Blum K, Chen AL, Giordano J (2012). The addictive brain: all roads lead to dopamine. *Journal of Psychoactive Drugs*.

[28] Slavich GM, Cole SW (2013). The emerging field of human social genomics. *Clinical Psychological Science*.

[29] Ducci F, Goldman D (2012). The genetic basis of addictive disorders. *Psychiatric Clinics of North America*.

[30] Otowa T, Gardner CO, Kendler KS, Hettema JM Parenting and risk for mood, anxiety and substance use disorders: a study in population-based male twins.

[31] Brent DA, Silverstein M (2013). Shedding light on the long shadow of childhood adversity. *Journal of the American Medical Association*.

[32] Nomura Y, Hurd YL, Pilowsky DJ (2012). Life-time risk for substance use among offspring of abusive family environment from the community. *Substance Use & Misuse*.

[33] Sirvinskiene G, Zemaitiene N, Zaborskis A, Markuniene E, Jusiene R (2012). Infant difficult behaviors in the context of perinatal biomedical conditions and early child environment. *BMC Pediatrics*.

[34] Fryers T, Brugha T (2013). Childhood determinants of adult psychiatric disorder. *Clinical Practice and Epidemiology in Mental Health*.

[35] Elton A, Tripathi SP, Mletzko T (2013). Childhood maltreatment is associated with a sex-dependent functional reorganization of a brain inhibitory control network. *Human Brain Mapping*.

[36] Flaherty EG, Thompson R, Dubowitz H (2013). Adverse childhood experiences and child health in early adolescenceJAMA Pediatrics.

[37] Spenhoff M, Kruger TH, Hartmann U, Kobs J (2013). Hypersexual behavior in an online sample of males: associations with personal distress and functional impairment. *The Journal of Sexual Medicine*.

[38] (2000). *Mental Health Care Services By Family Physicians (Position Paper)*.

[39] de Lepeleire J, Oud M, Buszewicz M (2012). Mental health problems in family medicine/general practice. *International Journal of Family Medicine*.

[40] Dawson GN, Warren DE (2012). Evaluating and treating sexual addiction. *American Family Physician*.

[41] Kaplan MS, Krueger RB (2010). Diagnosis, assessment, and treatment of hypersexuality. *Journal of Sex Research*.

[42] Chinman M, Oberman RS, Hanusa BH A Cluster randomized trial of adding peer specialists to intensive case management teams in the veterans health administration.

[43] Kotter T, Schaefer FA, Scherer M, Blozik E (2013). Involving patients in quality indicator development—a systematic review. *Patient Preference and Adherence*.

[44] Tierens E, Vandevoorde J, Devroey D Diagnosis and treatment of participants of support groups for hypersexual disorder in Belgium.

[45] Chong SA, Abdin E, Sherbourne C (2012). Treatment gap in common mental disorders: the Singapore perspective. *Epidemiology and Psychiatric Sciences*.

[46] Koski-Jännes A, Hirschovits-Gerz T, Pennonen M (2012). Population, professional, and client support for different models of managing addictive behaviors. *Substance Use and Misuse*.

[47] Schmittdiel JA, Shortell SM, Rundall TG, Bodenheimer T, Selby JV (2006). Effect of primary health care orientation on chronic care management. *Annals of Family Medicine*.

[48] Codony M, Alonso J, Almansa J (2009). Perceived need for mental health care and service use among adults in Western Europe: results of the ESEMeD project. *Psychiatric Services*.

[49] Donaldson MS, Vanselow NA (1996). The nature of primary care. *Journal of Family Practice*.

[50] Brohan E, Clement S, Rose D, Sartorius N, Slade M, Thornicroft G (2013). Development and psychometric evaluation of the Discrimination and Stigma Scale (DISC). *Psychiatry Research*.

[51] Corker E, Hamilton S, Henderson C (2013). Experiences of discrimination among people using mental health services in England 2008–2011. *The British Journal of Psychiatry*.

[52] http://www.aafp.org/.

[53] Crosson FJ (2009). 21st-Century health care—the case for integrated delivery systems. *The New England Journal of Medicine*.

[54] Adelson S, Bell R, Graff A (2012). Toward a definition of, “hypersexuality” in children and adolescents. *Psychodynamic Psychiatry*.

[55] Turner M (2008). Female sexual compulsivity: a new syndrome. *Psychiatric Clinics of North America*.

[56] Fergusson DM, McLeod GF, Horwood LJ (2013). Childhood sexual abuse and adult developmental outcomes: findings from a 30-year longitudinal study in New Zealand. *Child Abuse and Neglect*.

[57] Parsons JT, Grov C, Golub SA (2012). Sexual compulsivity, co-occurring psychosocial health problems, and HIV risk among gay and bisexual men: further evidence of a syndemic. *American Journal of Public Health*.

